# Lentil Sprouts Effect On Serum Lipids of Overweight and Obese Patients with Type 2 Diabetes

**DOI:** 10.15171/hpp.2015.026

**Published:** 2015-10-25

**Authors:** Zahra Aslani, Parvin Mirmiran, Beitollah Alipur, Zahra Bahadoran, Mahdie Abbassalizade Farhangi

**Affiliations:** ^1^Department of Nutrition, Faculty of Nutrition, Tabriz University of Medical Sciences, Tabriz, Iran; ^2^Nutrition and Endocrine Research Center, Research Institute of Endocrine Sciences, Shahid Beheshti University of Medical Sciences, Tehran, Iran

**Keywords:** Lentil sprouts, Triglyceride, Oxidized LDL cholesterol, Type 2 diabetes

## Abstract

**Background: ** The present study aimed to determine the effect of lentil sprouts [LS] on lipid profiles in overweight and obese patients with type 2 diabetes.

**Methods:** Forty- eight overweight and obese type 2 diabetic patients, September and November2013, 30-65 years, participated in this clinical trial and randomly divided into two groups; LS group and controls. Patients in control group received conventional drug therapy, while patients in LS group received 60 g LS daily during 8 weeks along with routine medication. Significant differences among and between the groups were determined by independent t-test and paired sample t-test using SPSS software. The patients were blinded for the treatment. In this trial the effect of LS on serum lipid profiles were inves-tigated.

**Results:** Thirty-nine patients completed the study. After 8 weeks, serum levels of HDL-C was higher in the LS group compared to control group (48.3 ±1.9 vs. 42.8±1.7, P<0.03). TG and ox-LDL were lower in the LS group compared to controls [(127±13.4 vs. 170± 12.4.P<0.01) and (83.3±29.1 vs. 98.7±28.2.P<0.6)].

**Conclusions: **LS consumption could have favorable effect on serum lipid profiles.

## Introduction

Type 2 diabetes is a metabolic disorder characterized by absolute or relative insulin deficiency, hyperglycemia, and disorder in carbohydrate as well as lipid metabolism.^1^“The development of chronic hyperglycemia in diabetes leads to severe damage in bodily tissues, organ dysfunctions and finally the irreversible failure of some critical organs of the body, especially eyes, kidneys, nerves and cardiovascular system”.^2^ Due to side effects of drugs used to treat diabetes, using complementary therapies and modification of the dietary pattern are good ways to improve the condition of disease. Legumes consumption has many effects in health improving, control and protection against metabolic diseases such as type 2 diabetes and cardiovascular diseases (CVD_s_).^3^ According to food ingredient table data, the amount of lentil seed fiber is 3.7 gr/100 and has a low glycemic index (21.1). After germination of seeds the amount of fiber and protein are increased.^4^ After consumption of lentils in diabetic patients TC and glucose decreasedsignificantly^5^, and after consumption of lentil sprouts (LS) glucose decreased.^6^ Besides, oxidized low density lipoprotein [ox-LDL] has a major role at the beginning and the development of atherosclerosis.^7^ HDL-C has a major role in inhibiting LDL-C oxidation particles.^8^

There are limited data regarding the potential properties of lentil sprouts on metabolic disorders; we therefore conducted this trial to investigate the effect of LS on serum TG, TC, LDL-C, HDL-C levels and atherogenic lipid parameters including AIP (atherogenic index of plasma), the ratios of ox-LDL/TC, ox-LDL/HDL-C and ox-LDL/LDL-C, TG/HDL and LDL/HDL were measured in overweight and obese patients with type 2 diabetes.

## Materials and Methods

### Study population 

Patients [male and female] with type 2 diabetes, referred to the Iran Diabetes Society and Endocrine Clinic of Taleghani Medical Center, Tehran, Iran were recruited for this study. Diagnosis of type 2 diabetes was according to American Diabetes Association.^9^ This trial was conducted between September and November 2013. The patients aged 30-65yr, with body mass index ranged 25-40 kg/m^2^ were included. Subjects with renal, hepatic disorders, gestation or lactation and patients who used insulin injection, contraceptive pills, glucocorticoid drugs or LS and multivitamin as well as supplements with antioxidant in the last 3 months were excluded from the study. Finally, forty-eight subjects [31 men and 17 women] were included and divided into two groups, LS=19 and control=20, randomly.

### Intervention

### Lentil sprouts preparation

The lentil seeds were placed in water for 30 hours. The seeds were placed on a wet cotton tissue. After 24 hours, lentil sprouts were packed and sent to patients. In these stages, temperature of environment was 25-30^°^C, but after the preparation, they were kept in a refrigerator.

### Treatment

Patients in LS group received 60 g LS daily, and the patients in control group continued regular diet. Patients in the LS group consumed LS alone or with salads fresh. According to Dietary Reference Intake, each patient consumed 60 g LS in LS group.^10^ In order to assess dietary changes during the study period three-day dietary recalls, including 2 weekends and 1 weekend day, was collected at baseline and again after 8 weeks from the subjects. Subjects provided with written informed consent. The method of consumption was written for the participants. They were asked to maintain their habitual life style, physical activity and dietary pattern during the eight weeks of study. Patients were excluded from the analysis if they consumed <85% of the packets^11^ or changed their medication or reported severe side effects. For being confident that the patients consume LS, the researcher called them weekly.

### Anthropometric and biochemical measurements

Anthropometric measurements including weight, height and waist circumference were measured at baseline and eight weeks later. Weight was measured with digital scale (Seca 707, Hamburg, Germany, nearest to 100g) while the subjects were minimally clothed without shoes. Height was measured to the nearest0.5 cm, in a standing position without shoes, using a tape meter. Body mass index [BMI] was calculated as follows: weight/ square of height [kg/m^2^]. Venous blood samples were drawn after an overnight fasting at baseline and again after intervention [Days 0 and 56] and were centrifuged at 4^°^C and 500 g for 10 min to separate plasma. Venous blood sample volume was 7 ml. Serum concentrations of TC, triglyceride [TG] and HDL-C were evaluated by using the standard enzymatic methods with commercially available Parsazmun kits (Tehran, Iran). LDL-C was calculated from serum TC, TG and HDL-C according to the friedewalde equation.^12^ TC was assayed with the cholesterol esterase and cholesterol oxidize method and TG was assayed by using glycerol phosphate oxidize. The monoclonal antibody was used by ELISA kit to qualify the concentration of serum oxidized LDL (Mercodia Company, Uppsala, Sweden). AIP as defined logarithm of the TG/HDL-C ratio, directly related to lipoprotein particle size and the risk of atherosclerosis [AS].^13,14^ Inter- Intra-assay coefficient of variations of all assays were <5%.

### Statistical analysis

According to statistical methods for triglyceride variable^15^ with 95% confidence and 80% power, the sample size with regard to possible loss of the samples was calculated 20 patients in each group. The experimental data were analyzed by SPSS software, version16 (SPSS Inc., Chicago, IL, USA). The normality of the distribution of variables was determined by the Kolmogorov– Smirnov test. Analysis of covariance was used to identify any differences between two groups after intervention, adjusted for baseline measurements and covariates including lipid lowering and anti- diabetic drugs. The changes in anthropometric measurements, lipid profile and ox-LDL parameters of the participants between the beginning and end of the trial were compared by paired sample *t*-tests. The percent change for each variable was also calculated by formula [(8- weeks values- baseline values)/baseline values×100]. Also, *P*- value <0.05 was considered significant. Main researcher generated the random, enrolled participants and assigned participants to interventions.

### Ethical approval

Ethics approval was obtained from Ethical Committee of Tabriz University of Medical Science. The trial has been registered in the Iranian Registry of Clinical trial at http://www.irct.ir with the following identification IRCT201305251640N9.

## Results

**Fig. 1:**
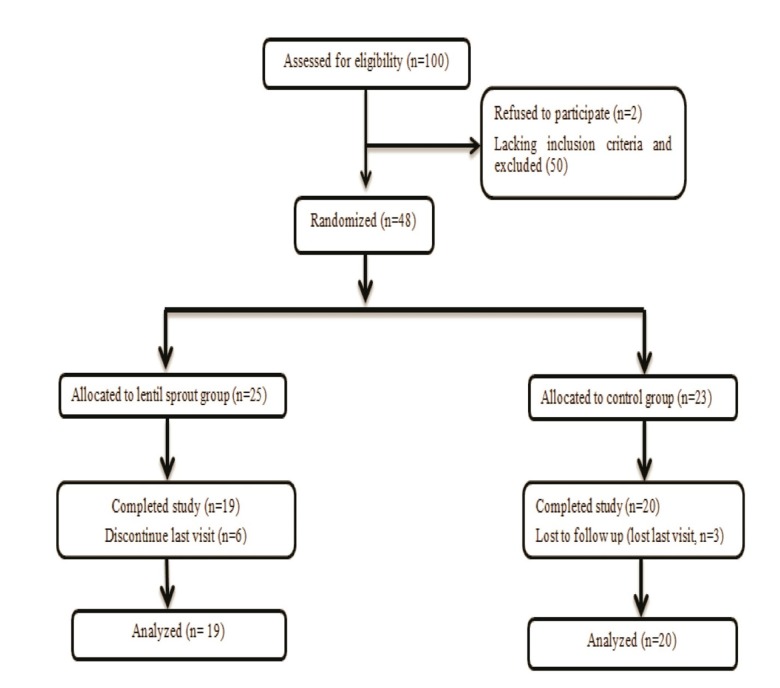
Flow chart of study participants

Forty-eight subjects were enrolled and divided into two groups randomly, but only 39 were available for data analysis because some of them could not complete the entire protocol or come for secondary evaluation. Final analysis was per protocol. Randomization of the patients was simply enveloped. Patients were blinded. These 39 patients were composed of 12 women and 27 men (Figure 1). Subjects’ demographics are shown in Table 1.

**Table 1:** Demographic characteristics of diabetic patients in Control and lentil sprout groups

**Variables**	**Control (n=20)**	**LS (n=19)**	***P***_value_
Age (yr)	54±7.4	52±7.6	0.9
Women/men(n)	7/13	5/14	0.5
Duration of diabetes(y)	7.4±6.6	11.7±19.2	0.3
Weight (kg)	78.5±10.5	78.3±9.8	0.7
Height (m)	1.63±0.09	1.66±0.08	0.6
BMI(kg/m^2^)	28.2±2.6	29.4±3.6	0.3
Glucoselowering drugs (n), (%)	(13), (65%)	(19), (100%)	0.01
Lipid lowering drugs (n)	2	9	0.03
Blood pressure lowering drugs(n)	3	7	0.1
FBS(mg/dl)	162.5±55	159±44	0.8

^a^ All values are mean±SD (unless stated otherwise).

No significant differences between groups were seen for mean age, duration of diabetes, weight, body mass index, blood lowering pressure drugs; nor were there any significant differences between the pretreatment serum values of total cholesterol, low density LDL-C, high density lipoprotein cholesterol [HDL-C], triglycerides, atherogenic index of plasma [AIP], TC/HDL-C ratio, LDL-C/HDL-C ratio, ox-LDL, ox-LDL/LDL-C ratio, ox-LDL/HDL-C ratio and ox-LDL/TC ratio in the 2 groups at baseline (Table2). Some patients used glucose lowering drugs including metformin and glibenclamide. Some participants used lipid-lowering drugs such as statins. Some of them used blood pressure lowering pressure such as nifdidin. Attention to disease severity differed amount of drugs for each patient.

**Table 2:** Biochemical values of diabetic patients in LS and control groups

**Variables**	**Control (n=20)**	**LS (n=19)**	***P***_value_
TC (mg/dl)	167.9±0.3	158.8±0.4	0.162
TG (mg/dl)	130±55.5	132±68.2	0.234
LDL-C (mg/dl)	97.5±27.8	88.9±34.5	0.383
HDL-C (mg/dl)	44.2±10.8	43.4± 11.8	0.935
AIP	0.05±0.015	0.051±0.016	0.451
TG/HDL-C ratio	3.2±2	3.3± 2.3	0.253
LDL-C/HDL-C ratio	2.3±0.8	2.2± 0.9	0.484
ox-LDL (mU/ml)	111±38.1	102.7± 60.5	0.215
ox-LDL/LDL-C ratio	1.3±0.7	1.5± 1.6	0.244
ox-LDL/HDL-C ratio	2.6±0.9	2.6± 1.8	0.862
ox-LDL/TC ratio	0.7±0.33	0.69± 0.5	0.402

TC, total cholesterol; TG, triglyceride; LDL-C, low density; HDL-C, high density lipoprotein; ox-LDL, oxidized low density lipoprotein; AIP; atherogenic index of plasma ^a^ All values are mean±SD.

There were significant differences in lipid lowering and anti-diabetic drugs between two groups, therefore these variables were adjusted in the analysis. The mean dietary intakes of participants at baseline and after 8-week of intervention a presented in Table 3.

**Fig. 2:**
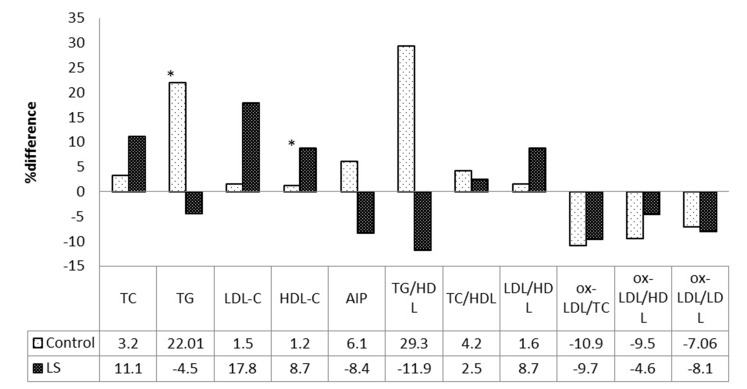
Mean differences of variables compared with baseline values in two groups (*significant difference within the groups using paired t-test, *P*<0.05).TC, total cholesterol; TG, triglyceride; LDL-C, low density lipoprotein; HDL-C, high density lipoprotein; ox-LDL, oxidized- low density lipoprotein; AIP, atherogenic index of plasma

There was no significant difference between the groups in total energy and nutrient intakes, as estimated by 3-day dietary recalls. Eight-week biochemical values of participants in the two groups, and the treatment effects of LS on lipid profiles are presented in Figure 2.

**Table 3:** Dietary intakes of the study participants at baseline and after 8 weeks of intervention in the two groups

**Variable**	**Control,(20)** **Mean (SD)**	**LS,(n=19)** **Mean(SD)**	**Mean difference** (95% confidence interval)*	***P*** *
**Energy (kcal)**				
Baseline	1894.4(464.3)	1892.5(427.4)	1.9(-95.14-229.13)	0.191
After intervention	1882.5(442.5)	1880.7(492.6)	2.02(-87.33-187.66)	0.163
Mean difference (95% confidence interval)**	-12.1(-142.5-111.3)	-11.1(-122-109)	-	-
P**	0.68	0.43	-	-
**Carbohydrate (g/d)**				
Baseline	249.4(69.2)	239.2(86.6)	10.2(-65.3-87.5)	0.357
After intervention	242.5(76.7)	226.3(64.5)	16.4(-77.2-43.6)	0.253
Mean difference (95% confidence interval)**	-7.1(-28-74)	-13.1(-64-35)	-	-
P**	0.54	0.63	-	-
**Protein(g/d)**				
Baseline	84(21.5)	76.1(28.2)	8(-3.1-17.3)	0.448
After intervention	86(26.3)	80.2(22.5)	6(-2.4-21.2)	0.363
Mean difference (95% confidence interval)**	2(-3-16)	4(-2-20)	-	-
P**	0.78	0.48	-	-
**Total fat (g/d)**				
Baseline	63.1(25.2)	64.3(28.5)	-1.2(-3.4-19)	0.287
After intervention	61.4(21.5)	62.8(28.1)	-1.4(-5.3-23)	0.334
Mean difference (95% confidence interval)**	-1.7(-4-21)	-1.5(-1.8-19)	-	-
P**	0.46	0.65	-	-
**Saturated fat(g/d)**				
Baseline	17.9(5.4)	18.3(6.2)	-0.4(-7.1-15.3)	0.631
After intervention	18.5(5.1)	19.4(6.2)	-0.9(-5.3-19	0.484
Mean difference (95% confidence interval)**	0.6(-4-23)	1.1(-3.3-25.2)	-	-
P**	0.55	0.43	-	-
**Mono-unsaturated fat(g/d)**				
Baseline	17.2(6.2)	18.4(7.5)	-1.2(-3.4-26.1)	0.458
After intervention	16.7(6.6)	17.8(8.1)	-1.1(-2.6-24.3)	0.563
Mean difference (95% confidence interval)**	-0.5(-4.1-22.3)	-04(-3.2-21.4)	-	-
P**	0.62	0.78	-	-
**Poly-unsaturated fat (g/d)**				
Baseline	15.7(7.4)	16.4(6.8)	-0.7(-4.6-22.4)	0.608
After intervention	15.1(7.1)	15.9(6.2)	-0.8(-2.6-18.5)	0.767
Mean difference (95% confidence interval)**	-0.6 (-3.2-17.4)	-0.5(-1.8-19.2)	-	-
P**	0.34	0.48	-	-
**Vitamin C(mg/d)**				
Baseline	57.8(30.8)	63.5(40.2)	-5.7(-4.2-25.1)	0.254
After intervention	67.8(36.8)	72.1(37.3)	-4.3(-2.8-28.4)	0.342
Mean difference (95% confidence interval)**	10(-1.9-23.4)	8.4 (-3.1-15.3)	-	-
P**	0.33	0.52	-	-
**Vitamin E(mg/d)**				
Baseline	4.1(6.6)	2.3(1.8)	1.8(-5.2-17.3)	0.47
After intervention	3.8(5.8)	2.5(2.3)	1.3(-4.2-18.1)	0.526
Mean difference (95% confidence interval)**	-0.3(-3.4-16.8)	-0.2(-3.7-18.2)	-	-
P**	0.44	0.35	-	-
**Total fiber (g/d)**				
Baseline	23.6(8.8)	23.7(6.5)	-0.1(-3-24)	0.454
After intervention	25.4(4.3)	24.2(7.7)	0.2(2.127.3)	0.378
Mean difference (95% confidence interval)**	1.8(-2.4-24.1)	0.5(-2.1-14.2)	-	-
P**	0.65	0.78	-	-

*Independent *t*-test, ** paired *t*-test

Data of this study were primary. In the LS group, the levels of HDL-C and LDL-C increased compared with baseline (*P*<0.03, *P*<0.06) but in the control group no significant change was seen. In the LS group the levels of TG, AIP and ox-LDL decreased compared with the baseline values (*P*<0.01 and *P*<0.07 and<0.6). Patients in the intervention group did not report any side- effects from LS consumption.

## Discussion

In this trial, we investigated the effect of LS on lipid profiles in overweight and obese patients with type 2 diabetes. TG and ox-LDL decreased (*P*<0.01 and *P*<0.6) and HDL-C increased (*P*<0.03) in LS group compared to control group.

Lipid blood disorders play a major role in CVD_s_ risk in diabetic patients.^16^ In an animal experiment, rats consumed whole cooked lentils, level of HDL-C was significantly increased.^17^ Low HDL-C is an independent cardiovascular risk factor and management of HDL-C may contribute to the optimization of cardiovascular risk in Type 2 diabetes.^18^ Changes in dietary pattern, physical activity and weight can convert HDL-C level.^19^ In this study, patients were asked not to change their dietary pattern and physical activity. Weight of patients did not differ significantly between 2 groups after 8 weeks intervention. In a study, a high-fiber diet improved glycemic control and reduced serum lipids and HDL-C level increased in LS group after 8 weeks of LS consumption and this effect could be due to some bioactive components in LS.^20^Lentil seeds are rich source of fiber and micronutrients. Picillium increased the level of serum HDL-C in patients with type 2 diabetes.^21^Hypertriglyceridemia is a common form of dyslipidemia frequently associated with coronary heart disease (CHD).^22^ Our results showed that TG level decreased in LS group after 8 weeks of LS consumption (*P*<0.01).Several mechanisms could explain favorable effects of lentil and lentil sprouts on lipid and lipoprotein metabolism; high content of fiber could decrease bile acid and cholesterol reabsorption from Ileum.^23^

Another important finding of the current study was decreased TG/HDL-C ratio following treatment with LS. Decreased TG /HDL ratio is related to large and non-atherogen LDL-C particles.^24,25^

Diet rich in fruits and vegetables due to plant fibers, carotenoids, anti-oxidants and phytosterols can prevent and control non-communicable diseases such as diabetes.^26^ Herbal antioxidants have insulin-like effects and increased glucose absorption in peripheral tissues.^27^ Lentil seeds are rich source of antioxidants as compared to other cereals and have high antioxidant capacity.^28^ Lentils had a higher oxygen radical absorbing capacity (ORCA)value than most of the common fruits and vegetables such as apples, plums, blackberries, cherries, figs, peaches, pears, orangs, garlic, cabbage and almonds.^29^Nutrient composition of lentil is shown in Table 4.^30^

**Table 4:** Nutrient composition of lentil

**Nutrient**	**Unit**	**Value per 100g**	**Cup 77g**
Proximates			
Energy	(kcal)	106	82
Protein	(g)	9	7
Total lipid	(g)	0.6	0.4
Carbohydrate, by difference	(g)	22	17
Minerals			
Iron, Fe	(mg)	3	2
Potassium, K	(mg)	322	248
Sodium, Na	(mg)	11	8
Vitamins			
Folat, DEF	(µg)	100	77
Vitamin C	(mg)	17	13
Niacin	(mg)	1.2	0.8

Low HDL-c and high TG concentration is considered as a main risk factor for development of, CHD.^31-33^ Plasma ox-LDL increases in conditions such as diabetes,^34^central obesity^35^ and CAD. This may be considered as a marker of the AS.^36^ High level of ox-LDL may indicate the severity of pathological inflammatory response.^37,38^ Oxidation ratio of LDL had a stronger correlation with CVD_s_ in comparison with plasma oxidized LDL.^39^ ox-LDL and oxidation ratio of LDL (ox-LDL/TC, ox-LDL/HDL-C and ox-LDL/LDL-C) were closely related with AS,^39^ and these indicators were better biomarkers for discriminating between patients with CVD_s_ and healthy subjects than the usual tests.^39^ ox-LDL/TC, ox-LDL/LDL-C, ox-LDL/HDL-C were higher in CVD_s_ group.^40^ Reduction of ox-LDL/LDL, ox-LDL/TC and ox-LDL/HDL-c could be related with key bioactive components in the LS.

The present study had a few limitations because the sample size was small and the study duration was 8 weeks. Further studies with longer duration and various doses may shed more light on importance of the therapeutic effects of LS in diabetic patients. Due to some inclusion and exclusion criteria such as specified BMI range defined for this study, our findings could not be generalized and interpreted to all type 2 diabetic patients.

## Conclusion

Consumption of LS as supplementary treatment in type 2 diabetes could have favorable effects on lipid metabolism and atherogenic lipid parameters. Such findings imply that nutritional intervention and use of functional foods such as lentil sprouts along with common medical treatment may be an important strategy in management of cardiovascular risk factors and metabolic disorders in type 2 diabetic patients.

## Competing Interests

There is no conflict of interests.

## Acknowledgements

This study was funded by the Faculty of Nutrition, Tabriz University of Medical Sciences and Research Institute of Endocrine Sciences of Shahid Beheshti University of Medical Sciences. The authors express appreciation to the participants of this study. The authors wish to thank Mrs. N. Shiva for critical editing of English grammar and syntax of the manuscript.
